# How much water can wood cell walls hold? A triangulation approach to determine the maximum cell wall moisture content

**DOI:** 10.1371/journal.pone.0238319

**Published:** 2020-08-31

**Authors:** Emil Engelund Thybring, Ramūnas Digaitis, Thomas Nord-Larsen, Greeley Beck, Maria Fredriksson

**Affiliations:** 1 Department of Geosciences and Natural Resource Management, Biomass Science and Technology, Forest Nature and Biomass, University of Copenhagen, Frederiksberg, Denmark; 2 Division of Building Materials, Department of Building and Environmental Technology, Lund University, Lund, Sweden; 3 Department of Geosciences and Natural Resource Management, Forest Resource Assessment and Bioenergy, Forest Nature and Biomass, University of Copenhagen, Frederiksberg, Denmark; 4 Department of Wood Technology, Norwegian Institute of Bioeconomy Research, Ås, Norway; Luleå University of Technology, SWEDEN

## Abstract

Wood is a porous, hygroscopic material with engineering properties that depend significantly on the amount of water (moisture) in the material. Water in wood can be present in both cell walls and the porous void-structure of the material, but it is only water in cell walls that affects the engineering properties. An important characteristic of wood is therefore the capacity for water of its solid cell walls, i.e. the maximum cell wall moisture content. However, this quantity is not straight-forward to determine experimentally, and the measured value may depend on the experimental technique used. In this study, we used a triangulation approach to determine the maximum cell wall moisture content by using three experimental techniques based on different measurement principles: low-field nuclear magnetic resonance (LFNMR) relaxometry, differential scanning calorimetry (DSC), and the solute exclusion technique (SET). The LFNMR data were furthermore analysed by two varieties of exponential decay analysis. These techniques were used to determine the maximum cell wall moisture contents of nine different wood species, covering a wide range of densities. The results from statistical analysis showed that LFNMR yielded lower cell wall moisture contents than DSC and SET, which were fairly similar. Both of the latter methods include factors that could either under-estimate or over-estimate the measured cell wall moisture content. Because of this and the fact that the DSC and SET methods are based on different measurement principles, it is likely that they provide realistic values of the cell wall moisture content in the water-saturated state.

## Introduction

The physical properties of wood depend to a large extent on the amount of moisture within the solid cell walls. It is therefore important to know the moisture content of the wood to predict the material performance. Under normal environmental conditions (temperature and relative humidity), moisture is predominantly found within the solid cell walls [1]. However, at high relative humidity (> 98–99%), moisture is also held as capillary water in the macro-void structure of the wood [2, 3]. To understand how moisture affects wood performance in the full moisture range, it is therefore important to be able to distinguish between cell wall water and capillary water in wood. Moreover, how much moisture that can be accommodated in the solid cell walls is an important characteristic of the material, i.e. the maximum cell wall moisture content. This moisture content is reached in the fully water-saturated state, when both cell walls and the wood void structure are saturated with water [1]. The maximum cell wall moisture content should, however, not be confused with the fibre saturation point (FSP) determined from changes in physical wood properties with moisture content [4], since the cell wall moisture content in this FSP state is lower than the maximum cell wall moisture content [1].

The total moisture content of wood can be experimentally determined with high accuracy using gravimetric techniques [5], however, classifying that moisture into cell wall water and capillary water by experimental methods is less straightforward. In the fully water-saturated state, there are three main techniques that can be used for this: Differential scanning calorimetry (DSC) [1, 6, 7], the Solute exclusion technique (SET) [8–11], and Low-field nuclear magnetic resonance relaxometry (LFNMR) [12–14]. However, because of the inherent measurement uncertainty in each of these techniques, it is difficult to accurately measure the maximum cell wall moisture content of wood. One way to tackle this issue is to use a large number of repeated measurements with a given technique. However, a given experimental technique may be biased towards either over- or under-estimation. The results may therefore be skewed towards higher or lower values than the true value, even if a large number of replicates are measured. Another way, which is perhaps more valuable, is to use a triangulation approach [15, 16], where the same parameter is determined using several techniques based on different measurement principles. Such an ensemble of experimental methods is typically associated with different underlying assumptions, uncertainties and biases. If agreement is found in the determined parameter across different techniques, this is a stronger indication that the measured value is close to the true value than relying on measurements from a single experimental method.

In this study, we used LFNMR, DSC, and SET, to determine the cell wall moisture content in the fully water-saturated state of specimens from nine different wood species. While these techniques have been used for a variety of wood species in previous studies, they have never been employed concurrently on the same sample material.

## Materials and methods

### Specimen preparation

Wood from nine different species with different densities was used in this study (Table 1). Oak, beech, and ash specimens originated from trees grown in Canton Zurich, Switzerland and was provided by colleagues at the Institute for Building Materials, ETH Zürich. The Norway spruce originated from an experimental forest in southern Sweden, for a detailed description, see [17] (mature sapwood, southern location). The poplar originated from southern Sweden (latitude: 55.85° longitude: 13.12°) and was felled in 2015 at an age of about 40 years. The spruce and poplar was kept in a climate room (20°C/60% relative humidity) for several years after felling. The remaining four species (abachi, balsa, Douglas fir, ironwood) were of unknown origin and had been kept in room climate for several years. The wood was cut into cubes with side 10 mm, dried in a vacuum oven at 60°C, and further extracted using a Soxhlet apparatus, first with ethanol and toluene (ratio 1:2) for 24 h and subsequently with acetone and MilliQ water (ratio 9:1) for 24 h. This was done since the extractives might otherwise interfere with the SET measurements. The density of the different wood species before and after extraction was determined for four of the cubes of each species. For these cubes, both the dry mass and the dimensions were determined after drying in a vacuum oven at 60°C before and after extraction. The mass was taken on a balance with 0.1 mg resolution and the dimensions were measured using a calliper with 0.01 mm resolution. For the latter, two measures were taken in each direction and the average of these was used to determine the volume of each cube. The dry densities before and after extraction were then determined as the dry masses divided by the dry volumes. The cubes were then further cut into samples sizes and geometries suitable for each method. The final specimen geometries were 5 x 10 x 10 mm^3^ (longitudinal x radial x tangential (L x R x T)) for SET, 10 x 5 x 5 mm^3^ (L x R x T) for LFNMR, and roughly circular discs of 4 mm diameter and 2 mm (L) thickness for DSC. The number of replicates for measurements with LFNMR and DSC was five, whereas it was three for SET. However, in the latter method two cuboids of 5 x 10 x 10 mm^3^ were used for each replicate measurement.

**Table 1:** Wood species used in this study along with their measured density before and after extraction. Species are listed after ascending density. Standard deviations are given in brackets.

Wood species	Botanical name	Density (kg m^-3^)	
		Initially	Extracted
Balsa	*Ochroma lagopus* Sw.	90 (9)	-
Abachi	*Triplochiton scleroxylon* K. Schum.	278 (24)	279 (32)
Poplar	*Populus x canadensis* Moench.	404 (13)	399 (17)
Norway spruce	*Picea abies* (L.) Karst.	405 (16)	387 (17)
Douglas fir	*Pseudotsuga menziesii* (Mirb.) Franco	577 (22)	539 (22)
Beech	*Fagus sylvatica* L.	606 (9)	572 (5)
Ash	*Fraxinus excelsior* L.	662 (9)	613 (4)
Oak	*Quercus robur* L.	751 (13)	695 (9)
Ironwood	*Lophira alata* Banks ex. Gartn.	1026 (13)	977 (9)

### Characterisation of the chemical composition

The chemical composition of the different wood species was determined by combined Thermogravimetric/Differential scanning calorimetry/Fourier transform infrared (TG/DSC/FTIR) analysis using a Netzsch STA 449F1 (Selb, Germany) combined with a Bruker Tensor FTIR (Billerica, MA, USA) [18–20]. The samples were ground to 1 mm mesh size and three replicates were analyzed for each wood species. Samples of 8 mg were weighed into Al_2_O_3_ crucibles and placed into the DSC/TG Octo S Type sample carrier together with an empty reference crucible. The samples were heated from 42°C to 710°C at a rate of 10°C min^-1^. Pure nitrogen gas (70 ml min^-1^) was used to purge the furnace from 42°C until 439°C, pyrolyzing the carbohydrate fraction of the material. Then the furnace gas was switched to a mixture of pure nitrogen (20 ml min^-1^) and air (50 ml min^-1^) to combust the remaining lignin and determine the ash content. FTIR spectra of the evolved gases were collected using 7 scans, providing an average spectrum every 6 seconds. The FTIR measurements were collected from 6000 cm^-1^ to 600 cm^-1^ with a resolution of 4 cm^-1^. Sixteen background scans were collected. A Gram-Schmidt (GS) curve was obtained from the acquired IR data. The GS orthogonalization allows a quantitative analysis of the total evolved gases detected by the spectrometer over time.

Chemical content was determined from sample mass loss at various temperature ranges. Extractive content was estimated from the mass loss between 105°C until the start of the first main peak in the GS curve. The hemicellulose fraction was obtained from mass loss from this point until a clear inflection point in either the first derivative of the GS curve or the first derivative of the DSC curve. Cellulose content was estimated from here until the next local minimum in the GS curve. Mass loss during the rest of the measurement was attributed to lignin. The mass remaining in the crucible after the measurement provided the ash content.

### Water-saturation and moisture conditioning

After extraction and final cutting of specimens, these were water-saturated by vacuum impregnation. This was done by placing the specimens of the same species of a given geometry in 50 mL reaction flasks. Thereafter, vacuum was applied for 30 minutes followed by injection of 20 mL MilliQ-water while pumping continued for 1 minute. Finally, atmospheric pressure was re-established after a period of 15 minutes without pumping.

### Determination of cell wall moisture content with low-field NMR relaxometry

LFNMR was used to distinguish between moisture in cell walls and different macro-voids within the wood specimens using a similar experimental procedure as described Fredriksson and Thygesen [12], but with settings as described below. The water-saturated specimens were measured one-by-one in a LFNMR probe (mq20-Minispec, Bruker, Billerica, MA, USA) held at constant temperature of 25°C. In the measurements, the spin-spin relaxation time (*T*_2_) was determined using a 1D Carr–Purcell–Meiboom–Gill (CPMG) pulse sequence [21, 22] with a pulse separation (*τ*) of 0.1 ms, 8000 echoes, 32 scans and a recycle delay of 30 s. For the specimens of abachi, ash, oak and ironwood, the total measurement time was not enough to give full decay of the LFNMR signal. For these specimens, the number of echoes was therefore increased to 20000. The 1D CPMG yields a decaying LFNMR signal which is then analysed by exponential decay analysis. This is done by fitting the decaying signal by the sum of one or more exponential functions with the fitting parameters being the characteristic decay time and the pre-exponential coefficient(s). Two types of exponential decay analysis were performed: discrete exponential and multi-exponential. In the previous, the “expfit” function (release version 1.5) [23, 24] for MATLAB was used. This function fits the decay signal with the sum of a selected number of exponentials. In this study, fitting was done with a range of 1 to 7 exponentials, and the optimum number of exponentials was then selected based on the residuals. These are reported by the “expfit” function as the norm of the vector in Euclidian space which contains the differences between fit and experimental data for the individual time points. With an increasing number of fitted exponentials, the residuals decreases but reaches a plateau after fitting around 3–5 exponentials. For each wood species, the optimum number of exponentials was then selected as the lowest number on the residuals plateau. For all wood species except Douglas fir and balsa, this optimum number of exponentials was four, while for Douglas fir and balsa it was three and five, respectively.

For multi-exponential decay analysis, the sum of a large number of exponentials is fitted to the decaying LFNMR signal. The characteristic decay times of these exponentials is logarithmically spaced in a pre-selected time interval which ranges from the first to the last time point in each data series. Thus, the aim of the analysis is to find the optimal combination of values for the pre-exponential coefficients that best fit the decay curve. The fitting was performed by use of the non-negative least squares algorithm of [25] which minimises the following statistic [26]:

χ2=∑j=1n(Ej−∑i=1NAie−tjτi)+1α∑i=1N(2Ai−Ai−1−Ai+1)2 (1)

where *A*_*i*_ (-) is the pre-exponential coefficient of the *i*^th^ relaxation component, *τ*_*i*_ (ms) is the characteristic time constant (*T*_2_ relaxation time) associated with the *i*^th^ relaxation component, *N* (-) is the number of exponential components fitted to the data, *E*_*j*_ (-) is the LFNMR signal in the *j*^th^ time point, *t*_*j*_ (ms) is the time of the *j*^th^ time point, *n* (-) is the total number of time points in the measurement series, and α (-) is a parameter that controls the smoothness of the spectrum, i.e. the difference in relative weights between adjacent *τ*’s in the selected range. In this study, 128 relaxation components (*N*) were fitted to the experimental decay curves, which resulted in a smooth spectrum with between 3 and 5 peaks, each representing water molecules in a specific physicochemical environment within the wood. The peak with the shortest *T*_2_ relaxation time, in the range of 1 ms, represents water molecules most tightly interacting with the solid wood material, i.e. the cell wall water [12, 27–33]. By multiplying the total moisture content with the sum of pre-exponential components *A*_*i*_ related to the peak with the shortest *T*_2_ relaxation time, and normalising this sum with the sum of all pre-exponential components of the peaks in the spectrum, a value for the fractional amount of cell wall moisture can be found. For the discrete exponential decay analysis, this quantity is determined by normalising the pre-exponential coefficient of the exponential with the shortest decay time with the sum of all pre-exponential coefficients determined. Subsequently, the fractional amount of cell wall moisture is multiplied by the total moisture content determined gravimetrically before the measurements to arrive at the cell wall moisture content, *u*_cw_ (g g^-1^) by

$$u_{cw}=u_{tot}\frac{S_{cw}}{S_{tot}}$$ (2)

where *u*_tot_ (g g^-1^) is the total moisture content of the wood, *S*_cw_ (-) is the pre-exponential coefficient or sum of pre-exponential coefficients related to cell wall water, and *S*_tot_ (-) is the sum of all pre-exponential coefficients *A*_*i*_ in the spectrum, i.e. excluding potential non-zero *A*_*i*_’s at each end of the spectrum found with multi-exponential decay analysis, since these are artefacts. In the statistical analysis, the results of the two analysis methods of the LFNMR data are treated as sub-methods, called LFNMR-discrete and LFNMR-multi.

### Determination of cell wall moisture content with differential scanning calorimetry

DSC was used to distinguish between water inside and outside of cell walls [1, 6, 34]. Each water-saturated specimen was placed in a sample pan (Tzero hermetic pans, TA Instruments, Eschborn, Germany) and a lid was put on which was then hermetically sealed with a Tzero press (TA Instruments, Eschborn, Germany). All DSC pans were then loaded in the autosampler of a DSC Q2000 (TA Instruments, Eschborn, Germany) and one-by-one measured in the following temperature cycle: First the pan was quenched to -20°C and held at isothermal conditions for 5 minutes before the temperature was increased by 2°C min^-1^ to 20°C. This cycle was repeated once before the temperature was finally increased rapidly from 20°C to 40°C and the sample pan unloaded and a new sample pan was loaded. After the DSC measurements, all lids were pierced multiple times with a syringe and the pans were dried in a vacuum-oven (65°C, 0 mbar) for 22 h. Finally, the dry masses were determined gravimetrically with a resolution of 0.01 mg after the dried pans had cooled over molecular sieves 3Å.

Heat flow curves from the experiments were analysed with the software TA Universal Analysis 2000 (version 4.5A, TA Instruments, Eschborn, Germany). The total melting energy, *Q* (J) was determined by integration of the melting peak in a temperature interval visually picked from the heating curves. The cell wall moisture content, *u*_cw_ (g g^-1^), was calculated for each specimen by

$$u_{cw}=\frac{m_{ws}-m_{dry}-(\frac{Q}{H_{f}})}{m_{dry}}$$ (3)

where *m*_ws_ (g) is the specimen mass in water-saturated state, *m*_dry_ (g) is the specimen dry mass, and *H*_f_ (J g^-1^) is the enthalpy of fusion of water of 333.7 J g^-1^. Calibration of the DSC Q2000 for enthalpy of fusion was done with deionised water (melting point 0°C, enthalpy of fusion 333.7 J g^-1^). In the statistical analysis, the results of the two temperature cycles in the DSC method are treated as two sub-methods.

### Determination of cell wall moisture content with solute exclusion

SET was used to determine the amount of moisture in water-saturated cell walls by probing the wood specimens with solute probe molecules too large to enter cell walls [8–11, 35]. Water-saturated wood specimens were added to individual 3.6 mL cryogenic nunc plastic vials after excess surface water was removed by dabbing each specimen on a water-soaked Wettex cloth (Wettex, Vileda, Freudenberg Home & Cleaning Solutions AB, Malmö, Sweden). About 1 cm^3^ wood was added to each vial. The mass of the wet specimens was then determined before 1.5 mL probe solution was added to each vial. The probe solutions consisted of a mix of polyethylene glycol molecules of different molecular masses and hence different sizes: PEG6k (average molar mass *M*_n_ = 6 000 g mol^-1^, hydrodynamic diameter *d* = 6.4 nm), PEG40k (*M*_n_ = 40 000 g mol^-1^, *d* = 17.9 nm), and PEG108k (*M*_n_ = 108 000 g mol^-1^, *d* = 30.5 nm). In order to have probe solution with narrow size distributions, PEG6k was acquired as reference standard from U.S. Pharmacopeia (USP, Rockville, MD, USA), while PEK40k and PEG108k were acquired as analytical standards for gel permeation chromatography from Sigma Aldrich (Darmstadt, Germany). Molecular sizes are according to regression of hydrodynamic diameter (*d*) with the molar mass (*M*_n_) by [36]. The initial concentration of each probe molecule was 0.6% w/w. After keeping the specimens in the probe solutions for 18 days at 5°C, the solution was removed with a 1 mL syringe by carefully pulling in the liquid from the top of the vial and then gradually tilting the vial while pulling the piston of the syringe. Hereby, the vast majority of the liquid in the vial was taken out. Subsequently, potential wood particles were removed by injecting the liquid into 2 mL HPLC glass vials through a nylon syringe filter (pore size 0.45 μm) put onto the tip of the syringe.

The quantification of probe molecules in the initial (stock) solution and the solution after specimen conditioning was determined with a Summit HPLC instrument (Dionex) equipped with Shodex OHpak SB-803 HQ with Phenomenex CHO-9225 guard column (0.18x250 mm tube between the two columns). Liquid samples were loaded in an autosampler set to 10°C, while the column oven and refractive index detector (Shodex) were kept at 40°C. The injection volume of analyte was 20 μL and the flow rate of the mobile phase (degassed MilliQ water) was kept at 0.8 mL min^-1^. Each sample measurement lasted 20 minutes and each liquid sample was measured 6–7 times (8 times for the stock solution), equally spaced over the several days it took to measure all liquid samples. The HPLC was controlled and analyte peak areas were calculated using the Chromeleon software (Thermo Fisher Scientific, Waltham, MA, USA). The borders of each peak were automatically assigned by the software, but all chromatograms were subsequently visually inspected and borders were corrected if deemed necessary. The average peak area of the multiple measurements on the liquid from a specific replicate was taken as the characteristic value for that replicate. In general, the repeated HPLC measurements showed a low variation in the calculated peak area with a coefficient of variation of 0.5% on average (max. 1.7%, min. 0.1%). Based on the change in peak area, i.e. concentration, between the initial state and after conditioning wood specimens, the cell wall moisture content, *u*_cw_ (g g^-1^) was determined as

$$u_{cw}=\frac{m_{ws}-m_{dry}-m_{sol}(\frac{c_{init}}{c_{\infty }}-1)}{m_{dry}}$$ (4)

where *m*_ws_ (g) is the specimen mass in water-saturated state, *m*_dry_ (g) is the specimen dry mass, *m*_sol_ (g) is the mass of the solution added, *c*_init_ (g L^-1^) is the initial concentration (peak area) of probe molecules in the solution, and *c*_*∞*_ (g L^-1^) is the concentration (peak area) of probe molecules after equilibrium has been attained. In the statistical analysis, the results obtained with the different PEG probe molecules in the SET method are treated as three sub-methods.

### Statistical analysis

Owing to the sampling design, where each repetition of measurements for a given tree species and method was taken from a single piece of wood, we expected probes taken from the same sample to be correlated. Consequently, the effect of the applied experimental method on the measured cell wall water content was analysed using a mixed linear model

$$y=X\beta +u+\varepsilon ,\;\mathrm{where}\;u∼N(0,G)\;\mathrm{and}\;\varepsilon ∼N\left(0,\sigma^{2}\right)$$ (5)

where *y* is the cell wall moisture content, ***X*** is the design matrix, **β** is a vector of fixed effects, **u** is a vector of random effects and ε is the random error. The mixed linear model was used for both analyses of the overall methods (LFNMR, DSC, and SET) and the various sub-methods using procedure MIXED in SAS v. 9.4 (SAS Institute, Cary, NC, USA). Multiple comparisons of individual molecular weights were analysed using Tukey-Kramer multiple comparisons procedure.

## Results and discussion

**Fig 1:**
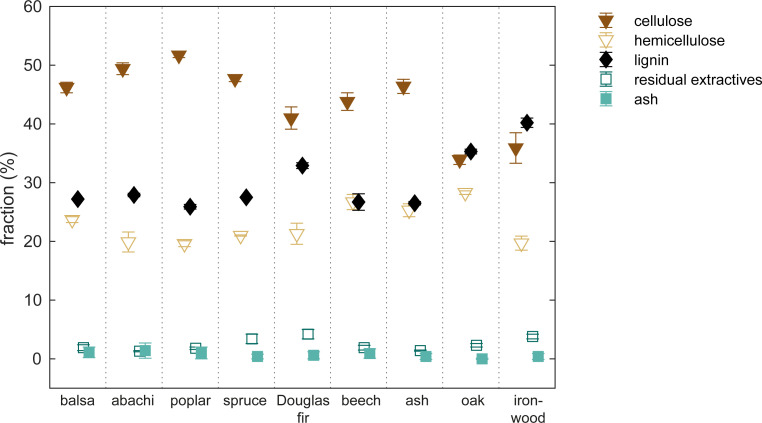
Chemical composition. Chemical composition of the nine wood species.

The density varied by more than one order of magnitude between the species with the highest (ironwood) and lowest (balsa) density, see Table 1. Of the nine wood species, the chemical composition of ironwood, oak, and Douglas fir differed from the rest in terms of a higher lignin content and lower cellulose content (Fig 1).

**Fig 2:**
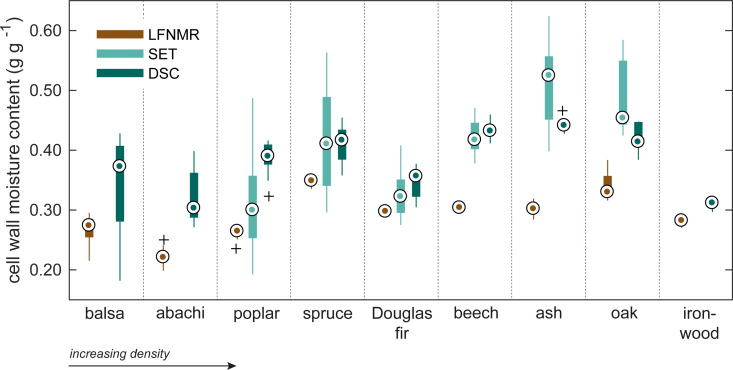
Cell wall moisture contents. Cell wall moisture contents determined with the three experimental techniques: low-field NMR relaxometry (LFNMR), differential scanning calorimetry (DSC), and the solute exclusion technique (SET). The central dot (.) indicates the median, the bottom and top edges of the box show 25^th^ and 75^th^ percentiles, respectively, and the whiskers extend to the extreme data points, while possible outliers are indicated by +. For SET, the results for abachi, balsa and ironwood are excluded from this plot because of excessive values determined for these species, see text for explanation.

The statistical analysis of the different methods on cell wall water content measurement first showed excess variance heterogeneity (Fig 2), i.e. the data variance differed excessively between the three methods. The reason was a very large variation of the SET measurements caused by extremely high cell wall water contents determined for balsa and abachi (Table 2). Moreover, for ironwood, the SET method yielded negative cell wall moisture contents for nearly all probe solutions. Possible reasons for this are discussed below. Further statistical analysis was therefore performed without balsa, abachi and ironwood.

**Table 2:** Mean cell wall moisture contents determined with the three experimental techniques: Low-field NMR relaxometry (LFNMR), differential scanning calorimetry (DSC), and the solute exclusion technique (SET).

	Maximum cell wall moisture content (g g^-1^)		
Wood species	LFNMR	DSC	SET
Balsa	0.266 (0.026)	0.337 (0.083)	5.367 (0.714)
Abachi	0.222 (0.014)	0.325 (0.046)	1.379 (0.242)
Poplar	0.262 (0.011)	0.386 (0.028)	0.308 (0.085)
Spruce	0.349 (0.007)	0.410 (0.030)	0.412 (0.083)
Douglas-fir	0.297 (0.005)	0.345 (0.026)	0.329 (0.039)
Beech	0.305 (0.004)	0.433 (0.015)	0.421 (0.029)
Ash	0.303 (0.010)	0.443 (0.010)	0.509 (0.074)
Oak	0.341 (0.021)	0.420 (0.024)	0.486 (0.056)
Ironwood	0.282 (0.006)	0.310 (0.007)	-0.050 (0.067)

Standard deviations are given in brackets.

In general, DSC and SET gave higher cell wall moisture contents than LFNMR, although the three sub-methods of SET and the two sub-methods of LFNMR differed. Statistical analysis of the three methods with the general linear model showed that LFNMR gave significantly lower (*P* < 0.001) cell wall moisture contents of about 0.1 g g^-1^ than DSC and SET. These latter two methods were, on other hand, not significantly different (*P* = 0.8675). However, the sub-methods produced significant different cell wall moisture contents for both the LFNMR (*P* < 0.01) and SET (*P* < 0.05) methods. In the previous, LFNMR-discrete yielded about 0.01 g g^-1^ lower values than LFNMR-multi, whereas the PEG108k yielded about 0.02 g g^-1^ and 0.08 g g^-1^ higher moisture contents than the PEG40k and PEG6k molecules, respectively. Of these latter two molecules, PEG40k resulted in cell wall moisture contents about 0.06 g g^-1^ higher than that found with PEG6k. In the DSC method, the first temperature cycle was found to give about 0.003 g g^-1^ lower cell wall moisture contents than the second cycle, however, this difference was not statistically significant. Further details and results concerning the statistical analysis can be found in S5–S12 Tables in S1 Appendix.

### Assumptions, uncertainties and biases of the experimental techniques

For successful use of the triangulation approach to determine the maximum cell wall moisture content, it is important to evaluate the underlying assumptions, uncertainties and potential biases of each of the experimental techniques. The uncertainties discussed relate to the assumptions of each method that may cause systematic errors (bias) in obtained data. An overview of the different methods with their assumptions, uncertainties and biases is given in Table 3.

**Table 3:** Descriptions, underlying assumptions and biased factors along with their potential effect, the probability, and the magnitude of error introduced in the determined cell wall moisture contents for the three methods used.

Method	Description	Assumptions	Bias	Probability and error
Low-field NMR relaxometry	• Measurement of a decaying low-field NMR signal • Signal data is fitted with a range of exponentials, where those with short relaxation time (~1 ms) represent cell wall water	• The characteristic pre-exponential coefficients derived by exponential decay analysis reflects the relative distribution of water in different environments	• Potential exchange of water molecules between environments during the measurement (under-estimation)	• Probability: High Error magnitude: Medium-Large
Differential scanning calorimetry	• Measurement of the heat flow during melting of ice in a frozen, water-saturated sample • Heat flow data is integrated in a range around 0°C to give the total energy required to melt the ice	• Cell wall water does not freeze/melt around 0°C • Energy for melting of the freezable water around 0°C represents capillary water	• Presence of non-freezable capillary water (over-estimation) • Presence of freezable cell wall water (under-estimation) • Non-saturation during measurement caused by temperature variation (under-estimation)	• Probability: High Error magnitude: Small • Probability: Negligible Error magnitude:- • Probability: Low Error magnitude: Small
Solute exclusion	• Measurement of the concentration change in a solution of probe molecules after soaking a water-saturated sample • Concentration data is collected with chromatography as the integral over the peak corresponding to a specific probe molecule	• All probe molecules are in solution • Probe molecules do not enter cell walls • At equilibrium, the concentration of probe molecules in solution within the wood structure is similar to that in the surrounding bulk solution	• Adsorption of probe molecules to solid material (under-estimation) • Penetration of cell walls by probe molecules (under-estimation) • Lower concentration within small voids than in the surrounding bulk solution (over-estimation)	• Probability: Medium Error magnitude: Medium-Large • Probability: Negligible Error magnitude: - • Probability: High Error magnitude: Small-Medium

The magnitude of error is categorised as being either small (< 0.01 g g^-1^), medium (0.01–0.05 g g^-1^), or large (> 0.05 g g^-1^). For further details about the error estimations please refer to the text.

### LFNMR relaxometry

Calculation of the cell wall moisture content from measurements with LFNMR is based on the assumption that the exponential decay analysis provides the relative distribution of moisture in different environments within the material. One uncertainty with this method is, however, that water molecules may exchange between the different water populations during the decay of the LFNMR signal in the experiment. This changes the distribution of relaxation times, since these water molecules will have relaxation times intermediate of water in those environments between which they are exchanging [37]. For instance, Beck et al. [14] used the 1D CPMG pulse sequence in both LFNMR measurements at 22°C and in cryo-conditions at -18°C to determine the cell wall moisture content. Beck et al. [14] found that the values obtained at 22°C were lower than the ones obtained at -18°C where the water in cell lumina is frozen and the exchange between cell walls and lumina is limited. The under-estimation at 22°C was 0.013–0.071 g g^-1^. In addition, data presented by Telkki et al. [32] indicates that cell wall moisture contents calculated from measurements at 14°C are lower than cell wall moisture contents calculated from measurements at -3°C. Furthermore, Valckenborg et al. [38] found that the pore size distribution of mortar derived from LFNMR measurements differed from that found by cryo-porosimetry; for the former, the total volume of the smaller pore sizes was under-estimated. Whether this was caused by exchanging water populations is not known. However, based on the above-mentioned results from literature it appears that the LFNMR performed above 0°C has a tendency to under-estimate the amount of water found in the smaller pore sizes, e.g. in the present study, water within the cell walls of wood.

The two types of exponential decay analysis did show differences in the determined cell wall moisture content of about 0.01 g g^-1^. Although this difference is statistically significant, the difference is small and indicates that the choice of analysis method of the LFNMR data only has a modest effect on the determined cell wall moisture content. However, potential differences between methods regarding the peaks representing water outside of cell walls were not evaluated in the present study. Agreements between discrete and multi exponential decay analysis methods have also been seen in previous studies of water in wood [12, 14].

#### Differential scanning calorimetry

Using DSC to determine the maximum cell wall moisture content relies on the assumption that the amounts of non-freezable and freezable water can be directly translated into cell wall water and capillary water, respectively. The presence of either freezable cell wall water or non-freezable capillary water would thus introduce an error in the determined cell wall moisture content. For the first case, if part of the cell wall water is freezable in the temperature range employed in the DSC measurement, the energy required to melt this will be recorded and be assigned to capillary water. Hereby, freezable cell wall water would cause an under-estimation of the cell wall moisture content. Since cell wall water is situated in very small cell wall pores of the order of 2 nm [39–41], it is expected to have a lower phase change (freezing/melting) temperature than liquid water [42, 43]. Although biopolymeric materials like extracted cellulose and lignin have been found to contain water which freezes well below the normal freezing point of liquid water [44, 45], this water is presumably found as clusters around strongly polar groups [46, 47]. No freezable cell wall water has, however, been found within solid wood samples [48, 49]. Therefore, the under-estimation in cell wall moisture content from freezable cell wall water is considered negligible.

Regarding the presence of non-freezable capillary water in the temperature range employed in the DSC measurement, such water would be assigned to cell wall water, hereby causing an over-estimation of the cell wall moisture content. Water close to solid surfaces can be restrained from freezing [50]. However, this layer of non-freezable water is typically in the range of 0.3–1 nm [51, 52], i.e. up to three water molecules thick. Yao and Ma [53] found that the visible internal surface area of loblolly pine determined by microscopy was around 9000 m^2^ per m^3^ wood for both early- and latewood tissue, despite the significant difference in density. A non-freezable water layer of 1 nm over this area corresponds with 90 cm^3^ water (i.e. about 90 g) per m^3^ wood. As the density varies between the investigated wood species, the resulting over-estimation is in the range 0.0001–0.001 g g^-1^ with an average value of 0.003 g g^-1^ for the wood species investigated. The internal surface area was also determined by Stamm and Millett [54] using both an adsorption method in a non-swelling solvent and microscopy. The two methods gave quite similar results in the range 0.22–0.25 m^2^ per gram wood. This results in a water volume of a 1 nm thick non-freezable layer of 0.0002 cm^3^ g^-1^, corresponding with a moisture content of 0.0002 g g^-1^. Even if the area estimations by Yao and Ma [53] and Stamm and Millett [54] are one order of magnitude too low, the over-estimation caused by the non-freezable capillary water is still less than 0.003 g g^-1^.

Another source of uncertainty in DSC measurements is the temperature changes occurring during the measurement itself which might change the maximum cell wall moisture content. It is well-known that the moisture content of wood depends on temperature, where an increasing temperature causes lower moisture contents in the hygroscopic range [55, 56]. However, very little data have been published regarding the temperature dependence of the cell wall moisture content near or at saturation. Stamm and Loughborough [57] showed that the fibre saturation point (FSP) decreases by 0.001 g g^-1^ per 1°C with increasing temperature. It is unclear how these FSP values were derived, but presumably they were based on extrapolation of sorption isotherm data. The FSP is, however, not the maximum cell wall moisture content [1], and these two parameters might not be similarly affected by temperature. For example, studies on cotton show that the temperature dependence of the sorption isotherm is different at low and high humidity levels; at low humidity levels, the moisture content decreased with increasing temperature, but at high temperatures, the opposite was seen [58]. Such behaviour would not be captured from extrapolation of sorption isotherms measured at low humidity levels. Only a single published study reports the maximum cell wall moisture content as function of temperature, although this was done on thermo-mechanically isolated pulp fibres [59]. Using solute exclusion with a dextran of high molecular mass, Eriksson et al. [59] found that the maximum cell wall moisture content increased steadily by 0.005 g g^-1^ per 1°C in the temperature range 20–70°C. Above 70°C, temperature had a more pronounced effect on the maximum cell wall moisture content which increased 0.014 g g^-1^ per 1°C as a result of lignin softening [59]. On the other hand, the maximum cell wall moisture content in lignin free fibres of cotton linters and holocellulose showed no temperature dependence. Although all these materials have a maximum cell wall moisture content considerably higher than that found for solid wood cell walls in this study, it appears likely that the maximum cell wall moisture content of wood is either constant or increases with increasing temperature.

For the DSC measurements, the wood is conditioned at 20°C, but is predominantly exposed to temperature beneath this value in the measurement itself. If considering only the 5 minutes at isothermal conditions and the temperature increase from -20°C to 20°C, the average temperature in the DSC measurement is -4°C. If moisture equilibrium was reached at this temperature and the maximum cell wall moisture content increased with 0.005 g g^-1^ per 1°C with increasing temperature, the DSC method would be expected to under-estimate the maximum cell wall moisture content at 20°C by 0.12 g g^-1^. However, it is necessary to consider the duration of the changes in temperature in order to estimate their effect on the maximum cell wall moisture content determined by the DSC method. Increasing the temperature from -20°C to 20°C takes 20 minutes, however, quenching the temperature the other way is more rapid as it takes about 3 minutes of which some time goes to stabilising the temperature at -20°C before the 5 minutes at isothermal conditions begin. During loading of the sample pan the temperature is 40°C for about 20–30 seconds before quenching commences. It is expected that the water kinetics slows down with decreasing temperature and is more or less locked below the freezing point, in particular for the frozen capillary water. In the worst case, the distribution between cell wall moisture and capillary water can shift for less than 2 minutes before the capillary water is frozen. However, during the temperature increase to 20°C the wood experiences 10 minutes above the freezing point. It is possible that some moisture redistribution occurs in the less than in total 12 minutes above 0°C. This redistribution can be evaluated by using two consecutive temperature cycles as in this study and comparing the obtained heat flow curves. In the present study, these two temperature cycles resulted in a difference in cell wall moisture content of 0.003 g g^-1^ on average, see S11 Table in S1 Appendix. Given the variability of the data, the difference is not found to be statistically significant, which indicates that the moisture distribution within specimens was not changed appreciably by quenching and re-heating on the time scale of the DSC experiment. The highest average difference in determined cell wall moisture content between the two temperature cycles was found for ash to 0.009 g g^-1^. Therefore, the under-estimation due to the changing temperature during the measurements is considered to be less than 0.01 g g^-1^.

#### Solute exclusion technique

Determination of the maximum cell wall moisture content by the SET method is based on the following assumptions:

1. after equilibrium is obtained all probe molecules are solubilised
2. probe molecules are only found in the void structure within the wood
3. there is an even concentration of probe molecules in the entire water volume accessible to them

Assumption 1 above would not be valid if the probe molecules fall out of solution, e.g. are adsorbed to the wood polymers lining the voids penetrated by the probes [9]. The equilibrium between the probe molecule solution inside the void structure and the solution surrounding the bulk specimen then changes. Since the concentration of probe molecules within the void structure will be decreased, more probe molecules will diffuse into the void structure. As a result, the change in solute concentration after exposure to water-saturated specimens is larger, which will result in the determined cell wall moisture content to be under-estimated.

Assumption 2 above is not valid if there are differences in penetration of various probe molecules into the material structure, e.g. if probe molecules are small enough to penetrate cell walls. This would result in various degrees of dilution of probe molecules penetrating cell walls and those exclusively penetrating the void structure. If probe molecules penetrate cell walls, the determined cell wall moisture content is under-estimated.

Lastly, assumption 3 above requires that the probe concentration in the solution surrounding the specimen is the same as the concentration within the accessible water volume in the wood. This assumption is not valid if the size of the accessible void or cell wall pore gets close to the size of the probe molecule [60–62]. For instance, if the probe molecules are half the size of the void they are penetrating, the probe concentration is only around 30% of that in the surrounding solution [60], and it diminishes further as the sizes of probe molecules and void get even closer. The result is that the probe solution is diluted less than if the concentration was even in the entire accessible water volume. This effect will therefore over-estimate the cell wall moisture content.

The probe molecules used in this study are PEG molecules of various sizes. They were chosen based on their relatively narrow size distribution. However, PEG molecules have a tendency to adhere to lignin [63] which is problematic due to assumption 1 above. This might be the reason why negative cell wall moisture contents were observed for ironwood for all sizes of PEGs, since ironwood has significantly higher lignin content than the rest of the wood species investigated, see Fig 1 and S1 Table in S1 Appendix. This indicates that the potential under-estimation caused by probe molecule adsorption can be large, since the LFNMR and DSC yield cell wall moisture contents that are 0.332–0.360 g g^-1^ higher than found with SET for ironwood.

**Fig 3:**
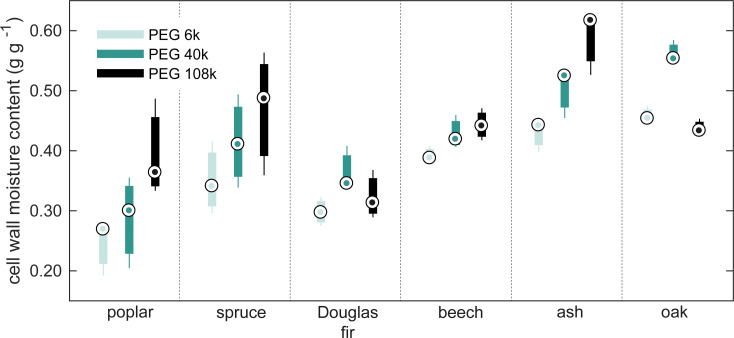
Cell wall moisture contents from SET measurements. Cell wall moisture content determined with the solute exclusion technique (SET) using three different sizes of polyethylene glycol (PEG) molecules. The central dot (.) indicates the median, the bottom and top edges of the box show 25^th^ and 75^th^ percentiles, respectively, and the whiskers extend to the extreme data points.

The three different PEG molecules did not yield similar maximum cell wall moisture contents as seen in Fig 3. In fact, statistical analysis of possible differences in cell wall water content measurements between the three PEGs showed significant differences (*P*<0.05), see S12 Table in S1 Appendix. The analysis furthermore showed that the measured cell wall moisture content increased with increasing molecular size. The size of the smallest PEG molecule used in the present study, PEG6k (*d* = 6.4 nm) is, however, well beyond the maximum pore size in water-swollen cell walls often reported to around 2–4 nm [39]. The largest PEG capable of penetrating cell walls appears to have a molecular weight of 3000–4000 g mol^-1^ [64, 65]. However, some studies report that even PEG20k penetrates the cell walls [66, 67], but this could be explained by the PEGs having broad distributions of molecular weights as shown by [65]. Since the PEG6k employed in this study was of analytical standard, it has a relatively narrow distribution of molecular weights spanning 5400–6600 g mol^-1^ according to the PEG6k material safety data sheet (USP, Rockville, MD, USA). Therefore, it seems unlikely that the differences in measured cell wall moisture contents with the various PEGs are caused by penetration of molecules on the lower tail of the size distribution for each PEG. The magnitude of error of this under-estimation is therefore considered negligible.

The probe molecules of this study range from a diameter of 6.4 nm to 30.5 nm, while the void structure of wood is on the micrometer scale. Even voids with a size of 1 μm are still 33–156 times larger than these probe molecules. Nonetheless, this size difference will cause a concentration in the void that is 1–6% lower than in the bulk solution, assuming a cylindrical geometry of the voids, see S4 Table in S1 Appendix. The LFNMR spectra provide a clue to how much water is confined in small macro voids. Thus, examination of the contributions from water with a *T*_2_ relaxation time around 10 ms gives an estimate of the fraction of water in small voids relative to the total water outside cell walls. This fraction varies markedly between the nine wood species; from 3.7% in poplar to 74.5% in ash (disregarding the data for abachi, balsa, and ironwood), see S3 Table in S1 Appendix. If the entire water volume described by this fraction is assumed conservatively to be confined to cylindrical pores of 1 μm diameter, the over-estimation can be calculated based on the partitioning coefficient *K* for the three probe molecules, see S1 Appendix. The results show that the over-estimation under these assumptions is 0.010 g g^-1^ (standard deviation 0.008 g g^-1^). The two species with the highest calculated over-estimations are ash and oak, which also contain the largest fraction of water in small voids outside cell walls of 74.5% and 58.4%, respectively. For these species the calculated over-estimation is 0.020 g g^-1^ for ash and 0.013 g g^-1^ for oak. At the same time, these two species are the only ones in which a significantly higher cell wall moisture content is found with SET than DSC. This indicates that the lower probe molecule concentration in small pores than in the bulk liquid may contribute to over-estimation in SET for some species with a large fraction of small macro voids, but is otherwise a modest effect.

A final source of bias in SET that is not related to the assumption given in Table 3 is a potential transport of water from the cell walls into the macro voids by osmosis. The concentration of probe molecules in the initial solution was 0.6% w/w for all probes, meaning that the smallest molecule has the highest molar concentration. This is the PEG6k probes with a molar concentration of 0.001 mol L^-1^ giving rise to an osmotic pressure of 2.3 mPa as calculated by the van’t Hoff formula. However, this small osmotic pressure essentially corresponds to a water activity of 1 and the osmosis effect for such low concentrations of relatively large molecules is thus negligible.

The variability in the obtained cell wall moisture content with different probe molecules highlights the importance of using multiple probe molecules with a size large enough to be excluded from the water-swollen cell wall porosity. Additionally, probe molecules with lower affinity for lignin could be used, e.g. dextrans [59, 68–70]. The extremely high cell wall moisture contents and large spread observed for balsa and abachi could likely derive from their low density and hence low specimen mass (0.12–0.38 g) since the same volume of material was used for all wood species. Thus, SET might not be suitable for species of very low density because of too low cell wall mass to void water volume.

## Conclusion

Statistical analysis of the obtained data showed that LFNMR gave lower cell wall moisture contents than those obtained with DSC and SET. It seems as if LFNMR measurements above 0°C leads to an under-estimation of the measured value, possibly due to an exchange between different pools of water. However, both DSC and SET include factors that either under-estimates or over-estimates the measured cell wall moisture content and these errors are potentially smaller than the underestimation obtained by LFNMR. In addition, these two methods are based on different principles of measurement, but still gave similar results. It is therefore likely that these methods provide realistic values of the cell wall moisture content in the water-saturated state. However, for SET, there are some limitations in terms of which wood species the method is suitable for.

## Supporting information

S1 Data DSC data Part 1. Heat flow data from Differential scanning calorimetry for abachi, ash, balsa, beech and Douglas fir.
(XLSX)

S2 Data DSC data Part 2. Heat flow data from Differential scanning calorimetry for ironwood, Norway spruce, oak, poplar, and water controls.
(XLSX)

S3 Data DSC data Part 3. Sample masses, evaluated moisture contents etc. for all samples included in the Differential Scanning calorimetry experiments.
(XLSX)

S4 Data LFNMR data. Data from Low field nuclear magnetic resonance measurements.
(XLSX)

S5 Data SET data. Data from measurements with the Solute exclusion technique.
(XLSX)

S1 Appendix Additional details on evaluation and experimental procedures. Further information on differences between different evaluation procedures and choices made in experimental and evaluation procedures.
(PDF)
