# Supplementary material for: How much water can wood cell walls hold? A triangulation approach to determine the maximum cell wall moisture content
Source: PLoS One. 2020 Aug 31;15(8):e0238319. doi: 10.1371/journal.pone.0238319 (PMC7458317; doi:10.1371/journal.pone.0238319)
Supplement: S1 Appendix — Further information on differences between different evaluation procedures and choices made in experimental and evaluation procedures. (PDF) [file pone.0238319.s006.pdf]

S1 Appendix. Additional details on evaluation and experimental procedures. Further information on differences between different evaluation procedures and choices made in experimental and evaluation procedures.

Supporting Information to

How much water can wood cell walls hold? A triangulation approach to determine the maximum cell wall moisture content

Emil Engelund Thybring[1], Ramunas Digaitis[1,2,#a], Thomas Nord-Larsen[3], Greeley Beck[4], Maria Fredriksson[2]*

*corresponding author (maria.fredriksson@byggtek.lth.se)

[1] Biomass Science and Technology, Forest Nature and Biomass, Department of Geosciences and Natural Resource Management, University of Copenhagen, Frederiksberg, Denmark

[2] Division of Building Materials, Department of Building and Environmental Technology, Lund University, Lund, Sweden

[3] Forest Resource Assessment and Bioenergy, Forest Nature and Biomass, Department of Geosciences and Natural Resource Management, University of Copenhagen, Frederiksberg, Denmark

[4] Department of Wood Technology, Norwegian Institute of Bioeconomy Research, Ås, Norway

[#a] Current Address: Biomedical Laboratory Science and Technology, Faculty of Health and Society, Malmö University, Malmö, Sweden

Chemical composition analysis

Table S1 Results from chemical composition analysis using TG/DSC/FTIR. Note that this analysis was done after extraction and the extractives are thus residual extractives not removed by the extraction process.

Wood species	Chemical composition (%)				
	Cellulose	Hemicelluloses	Lignin	Residual extractives	Ash
Balsa	46.2 (0.9)	23.7 (0.5)	27.2 (0.1)	1.9 (0.5)	1.1 (0.9)
Abachi	49.4 (1.0)	19.9 (1.7)	27.9 (0.3)	1.3 (0.1)	1.4 (1.3)
Poplar	51.7 (0.4)	19.6 (0.5)	25.9 (0.4)	1.8 (0.2)	1.0 (1.0)
Norway spruce	47.7 (0.5)	21.0 (0.1)	27.5 (0.1)	3.4 (0.8)	0.4 (0.4)
Douglas fir	41.0 (1.9)	21.3 (1.8)	32.9 (0.5)	4.2 (0.8)	0.6 (0.6)
Beech	43.8 (1.5)	26.7 (1.3)	26.7 (1.4)	1.9 (0.4)	0.9 (0.8)
Ash	46.4 (1.2)	25.3 (1.1)	26.5 (0.3)	1.4 (0.1)	0.4 (0.5)
Oak	33.9 (0.8)	28.3 (0.3)	35.3 (0.4)	2.3 (0.3)	0.0 (0.1)
Ironwood	35.9 (2.6)	19.7 (1.2)	40.2 (0.8)	3.8 (0.4)	0.4 (0.5)

Low-field NMR relaxometry: Effect of partial vs. full relaxation of signal

For the wood species abachi, ash, ironwood, and oak, it was not enough to use 8000 echoes with a τ of 0.1 ms to obtain full decay of the LFNMR signal. This is illustrated in Fig S1 for the measurements on ash and oak, where the lack of full decay is seen to affect particularly the peaks with the longest relaxation time. The peak with the shortest relaxation time reflects the cell wall water and it is less affected in terms of the relaxation time (location of peak top on x-axis). However, the relative weight of the cell wall water peak

does change between the partially and fully decayed curves as reflected in the determined cell wall moisture content (Table S1).

Table S2 Cell wall moisture content determined for partially (8000 echoes) and fully (20000 echoes) decayed LFNMR curves for abachi, ash, ironwood, and oak. Evaluated with multi-exponential decay analysis (LFNMR-multi).

	Echoes	Abachi	Ash	Ironwood	Oak
Cell wall moisture content (g g^{-1})	8000	0.225 (0.008)	0.310 (0.001)	0.283 (0.003)	0.332 (0.002)
	20000	0.218 (0.013)	0.302 (0.002)	0.286 (0.004)	0.332 (0.003)

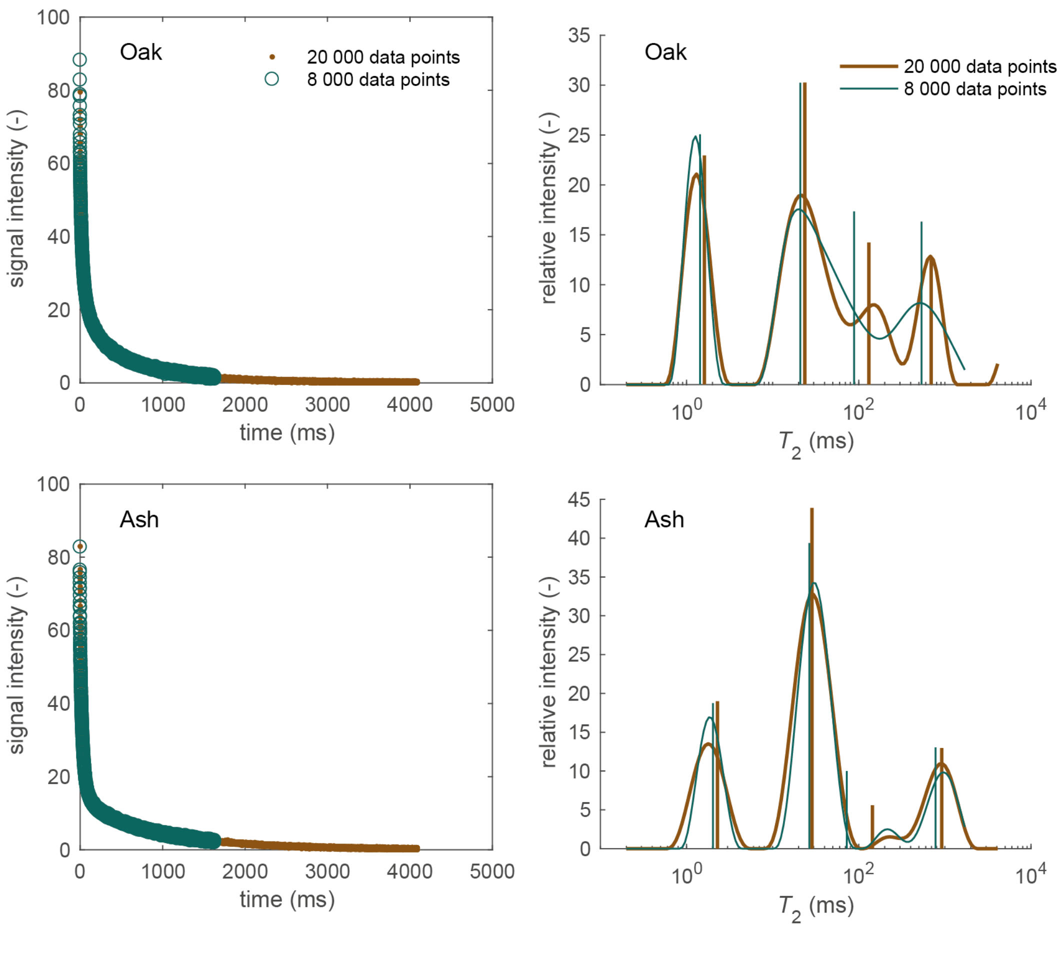

Fig S1. LFNMR relaxation curves (left) with 8000 and 20000 echoes and the resulting relaxation spectra (right) for the wood species oak (upper row) and ash (bottom row).

Low-field NMR relaxometry: Fraction of moisture in small voids

Based on the distribution of T_2 relaxation times obtained from the multi-exponential decay analysis of Low-field NMR relaxometry data, the fraction of moisture in small voids (i.e. with T_2 relaxation times around 10 ms) out of the total moisture outside cell walls was determined, see Table S3. This was calculated as fraction of the sum of the pre-exponential coefficients designating to the peak(s) around 10 ms to the sum of pre-exponential coefficients designating all peaks with longer T_2 relaxation times than the cell wall water peak.

Table S3. Fraction of moisture in small voids relative to the total amount of moisture outside cell walls based on T_2 relaxation spectra from multi-exponential decay analysis of low-field NMR relaxometry data. Standard deviations based on five replicates for each species are given in brackets.

Wood species	Fraction (-)
Balsa	0.055 (0.008)
Abachi	0.006 (0.006)
Poplar	0.037 (0.032)
Norway spruce	0.080 (0.005)
Douglas fir	0.206 (0.040)
Beech	0.272 (0.008)
Ash	0.745 (0.013)
Oak	0.584 (0.020)
Ironwood	0.445 (0.134)

Differential scanning calorimetry: Uncertainty in picking melting peak borders

Determining the cell wall moisture content with the DSC method involves visually picking the borders of the melting peak in each heating curves. This entails some uncertainty since the peak is broad rather than sharply defined. To evaluate the uncertainty of visually picking the peak border, the data for the first replicate of each wood species was selected and duplicated three times. Thus, for each wood species four identical heating curves were obtained. In total the data comprised of 36 heating curves consisting of nine sets of identical curves. The border of the melting peak on each of these curves was then picked by evaluating one curve for each wood species before the next duplicate was evaluated and so on. This was done instead of evaluating all four heating curves for one wood species in sequence, since this could have caused memorisation of the peak borders in the identical heating curves. By shuffling the heating curves this effect was avoided. The standard error was found to 0.008 g g^{-1} with a maximum deviation from the average cell wall moisture content of less than 0.03 g g^{-1}. This evaluation procedure was therefore considered to be robust.

Solute exclusion: Determination of appropriate equilibration times

A fundamental requirement of SET is that the probe molecule concentration is determined after equilibrium between the water within the wood and the surrounding solution. Enough time should be allowed for equilibrium to establish before the probe solution is removed. In order to investigate the appropriate equilibration time a pre-study with Norway spruce was undertaken using glucose, cellobiose, and PEG200 as probe molecules. These molecules are significantly smaller than the PEG6k, PEG40, and PEG108k utilised in the main study, and are able to penetrate the water-saturated wood cell walls. This gives them a longer expected time to full equilibration than the large PEG molecules, since glucose, cellobiose and PEG200 diffuse into both the macro-void structure and the nano-porous cell walls. By determining the apparent cell wall moisture content as function of the equilibration time, we therefore arrive at a conservative estimate for the appropriate time to equilibrate the wood and probe solutions. From Fig S2 it appears that after about 4 days, the Norway spruce samples and surrounding probe solution had reached equilibrium. However, given that the macro-void structure is variable between wood species, an excessive equilibration time of 18 days was selected in the main study.

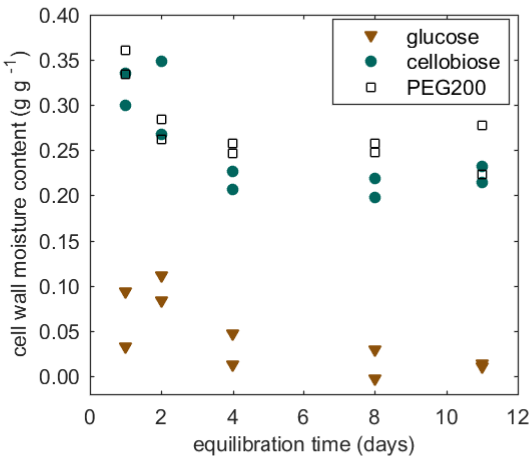

Fig S2 Determined cell wall moisture content of Norway spruce (*Picea abies* (L.) Karst.) for the three probe molecules glucose, cellobiose, and PEG200 as function of the equilibration time.

Solute exclusion: Effect of small voids in the macro-void wood structure

The calculation of the cell wall moisture content by Eq. 4 of the main manuscript assumes that the probe concentration in all the water accessible to the probes is the same and equal to the concentration in the surrounding bulk solution. As discussed in the manuscript this is incorrect, since the concentration of probes in water found in narrow voids is lower than in the bulk solution (Casassa 1967; Dai et al. 1998; Day et al. 1979; Dubin et al. 1993). The ratio between the probe concentration within the void and in the surrounding bulk solution, *K* (-), is called the “distribution coefficient”, “partitioning coefficient” or “exclusion coefficient” in literature (Yao and Lenhoff 2004). For simple pore geometries, the coefficient can be determined by

$$K = \frac{c_{\text{void}}}{c_{\text{bulk}}} = \left(1 - \frac{d}{q}\right)^n \qquad \text{(Eq. S1)}$$

where c_{void} (g L^{-1}) is the probe concentration in the void, c_{bulk} (g L^{-1}) is the probe concentration in the bulk solution, *d* (m) is the probe size, *q* (m) is the void size, and *n* is an exponent depending on the pore geometry; for slit voids *n* = 1, for cylindrical voids *n* = 2, and for spherical voids *n* = 3 (Casassa 1967; Casassa and Tagami 1969; Dai et al. 1998). Table S2 shows the reduction in the concentration of the three PEG probe molecules in voids of 1 µm (1000 nm) size.

Table S4. Reduction in probe concentration in 1 µm voids for the three PEG probe molecules employed in the study.

	PEG108k	PEG40k	PEG6k
Probe size, *d* (nm)	30.5	17.9	6.4
Void size, *q* (nm)	1000	1000	1000
**Slit voids**			
K_{slit} (-)	0.970	0.982	0.994
Reduction in probe concentration (%)	3.1	1.8	0.6
**Cylindrical voids**			
K_{cylinder} (-)	0.940	0.965	0.987
Reduction in probe concentration (%)	6.0	3.5	1.3
**Spherical voids**			
K_{sphere} (-)	0.911	0.947	0.981
Reduction in probe concentration (%)	8.9	5.3	1.9

Statistical analysis: Evaluation of differences between experimental methods

When all data were analysed for all wood species, there were significant differences ($P < 0.001$) between the three experimental methods (Table S3). However, the residuals for abachi and balsa were large and the SET-values determined for ironwood were negative, i.e. nonsensical. Therefore, the data for these three wood species were excluded from further statistical analysis.

For the remaining six wood species, the results from DSC and SET are not significantly different ($P = 0.8675$), see Table S4. For LFNMR, on the other hand, was found to give significantly lower ($P < 0.001$) cell wall moisture contents of about 0.1 g g^{-1} than the other two experimental methods.

If the SET is only represented by one of the three PEG molecules used, all three methods are found to be significantly different from each other ($P < 0.05$), see Tables S5-S7. The PEG108k molecule results in a cell wall moisture content determined by SET that is about 0.04 g g^{-1} higher than found with DSC (Table S5). The PEG40k molecule results in about 0.02 g/g higher cell wall moisture contents than found with DSC (Table S6), while the PEG6k molecule results in about 0.04 g/g lower cell wall moisture contents than found with DSC (Table S7).

Table S5 Differences of least squares means for all three methods on all nine wood species.

Method 1	Method 2	Estimate of diff. (g g^{-1})	Standard error (g g^{-1})	P-value, adjusted
DSC	LFNMR	0.08685	0.02290	0.0006
DSC	SET	-0.6391	0.02352	< 0.0001
LFNMR	SET	-0.7260	0.02352	< 0.0001

Table S6 Differences of least squares means for all three methods. Data for abachi, balsa, and ironwood have been excluded from the statistical analysis.

Method 1	Method 2	Estimate of diff. (g g^{-1})	Standard error (g g^{-1})	P-value, adjusted
DSC	LFNMR	0.09676	0.008262	< 0.0001
DSC	SET	-0.00448	0.008818	0.8675
LFNMR	SET	-0.1012	0.008818	< 0.0001

Table S7 Differences of least squares means for all three methods. Data for abachi, balsa, and ironwood have been excluded from the statistical analysis. For the SET method only the sub-method PEG108k is included.

Method 1	Method 2	Estimate of diff. (g g^{-1})	Standard error (g g^{-1})	P-value, adjusted
DSC	LFNMR	0.09676	0.008080	< 0.0001
DSC	SET-PEG108k	-0.03670	0.009489	0.0008
LFNMR	SET-PEG108k	-0.1335	0.009489	< 0.0001

Table S8 Differences of least squares means for all three methods. Data for abachi, balsa, and ironwood have been excluded from the statistical analysis. For the SET method only the sub-method PEG40k is included.

Method 1	Method 2	Estimate of diff. ($g\ g^{-1}$)	Standard error ($g\ g^{-1}$)	P-value, adjusted
DSC	LFNMR	0.09676	0.007187	< 0.0001
DSC	SET-PEG40k	-0.02071	0.008478	0.0457
LFNMR	SET-PEG40k	-0.1175	0.008478	< 0.0001

Table S9 Differences of least squares means for all three methods. Data for abachi, balsa, and ironwood have been excluded from the statistical analysis. For the SET method only the sub-method PEG6k is included.

Method 1	Method 2	Estimate of diff. ($g\ g^{-1}$)	Standard error ($g\ g^{-1}$)	P-value, adjusted
DSC	LFNMR	0.09676	0.005768	< 0.0001
DSC	SET-PEG6k	0.04396	0.006881	< 0.0001
LFNMR	SET-PEG6k	-0.05280	0.006881	< 0.0001

Statistical analysis: Evaluation of differences between sub-methods

The statistical analysis of differences between sub-methods within each experimental method was performed without the data of abachi, balsa, and ironwood. For the LFNMR sub-methods, a significant difference ($P < 0.01$) was found between DISCRETE and MULTI with the latter resulting in about 0.01 $g\ g^{-1}$ lower cell wall moisture contents than the previous (Table S8). The two temperature cycles in the DSC method was found to result in a cell wall moisture content that was about 0.003 $g\ g^{-1}$ lower in the first temperature cycle. However, this difference was found not to be statistically significant ($P = 0.0757$), see Table S9. All three probe molecules in the SET method were found to give statistically significant differences in the determined cell wall moisture content (Table S10). The PEG108k molecule resulted in a higher cell wall moisture content of about 0.02 $g\ g^{-1}$ ($P = 0.0472$) and 0.08 $g\ g^{-1}$ ($P < 0.001$) than those found with the PEG40k and PEG6kmolecules, respectively. Of these latter two molecules, the PEG40k molecule results in a cell wall moisture content that is about 0.06 $g\ g^{-1}$ ($P < 0.001$) higher than that found with the PEG6k molecule.

Table S10 Differences of least squares means for the two sub-methods of LFNMR. Data for abachi, balsa, and ironwood have been excluded from the statistical analysis.

Sub-method 1	Sub-method 2	Estimate of diff. ($g\ g^{-1}$)	Standard error ($g\ g^{-1}$)	P-value, adjusted
DISCRETE	MULTI	0.009648	0.002761	0.0015

Table S11 Differences of least squares means for the two sub-methods of DSC. Data for abachi, balsa, and ironwood have been excluded from the statistical analysis.

Sub-method 1	Sub-method 2	Estimate of diff. ($g\ g^{-1}$)	Standard error ($g\ g^{-1}$)	P-value, adjusted
Cycle 1	Cycle 2	-0.00331	0.001798	0.0757

Table S12 Differences of least squares means for the three sub-methods of SET. Data for abachi, balsa, and ironwood have been excluded from the statistical analysis.

Sub-method 1	Sub-method 2	Estimate of diff. ($g\ g^{-1}$)	Standard error ($g\ g^{-1}$)	P-value, adjusted
PEG108k	PEG40k	0.01599	0.006332	0.0472
PEG108k	PEG6k	0.08067	0.006332	< 0.0001
PEG40k	PEG6k	0.06468	0.006332	< 0.0001

References

Casassa EF (1967) Equilibrium distribution of flexible polymer chains between a macroscopic solution phase and small voids. Journal of Polymer Science Part B: Polymer Letters 5:773-778 https://doi.org/10.1002/pol.1967.110050907

Casassa EF, Tagami Y (1969) An equilibrium theory for exclusion chromatography of branched and linear polymer chains. Macromolecules 2:14-26 https://doi.org/10.1021/ma60007a003

Dai H, Dubin PL, Andersson T (1998) Permeation of small molecules in aqueous size-exclusion chromatography vis-à-vis models for separation. Analytical Chemistry 70:1576-1580 https://doi.org/10.1021/ac970968m

Day JC, Alince B, Robertson AA (1979) The characterization of pore systems by macromolecular penetration. Cellulose Chemistry and Technology 13:317-326

Dubin PL, Edwards SL, Mehta MS, Tomalia D (1993) Quantitation of non-ideal behavior in protein size-exclusion chromatography. Journal of Chromatography A 635:51-60 https://doi.org/10.1016/0021-9673(93)83113-7

Yao Y, Lenhoff AM (2004) Determination of pore size distributions of porous chromatographic adsorbents by inverse size-exclusion chromatography. Journal of Chromatography A 1037:273-282 https://doi.org/10.1016/j.chroma.2004.02.054
